# *Campylobacter jejuni* Cas9 Modulates the Transcriptome in Caco-2 Intestinal Epithelial Cells

**DOI:** 10.3390/genes11101193

**Published:** 2020-10-14

**Authors:** Chinmoy Saha, Deborah Horst-Kreft, Inez Kross, Peter J. van der Spek, Rogier Louwen, Peter van Baarlen

**Affiliations:** 1 Department of Medical Microbiology and Infectious Diseases, Erasmus MC University Medical Center Rotterdam, 3015 CN Rotterdam, The Netherlands; d.kreft@erasmusmc.nl (D.H.-K.); i.v.kross@umcutrecht.nl (I.K.); r.louwen@erasmusmc.nl (R.L.); 2 Department of Pathology and Clinical Bioinformatics, Erasmus MC, University Medical Center Rotterdam, 3015 GD Rotterdam, The Netherlands; p.vanderspek@erasmusmc.nl; 3 Host–Microbe Interactomics, Wageningen University and Research, 6708 WD Wageningen, The Netherlands; peter.vanbaarlen@wur.nl

**Keywords:** *Campylobacter jejuni*, Cas9, Caco-2, cell death, p53, NF-κB

## Abstract

The zoonotic human pathogen *Campylobacter jejuni* is known for its ability to induce DNA-damage and cell death pathology in humans. The molecular mechanism behind this phenomenon involves nuclear translocation by Cas9, a nuclease in *C*. *jejuni* (CjeCas9) that is the molecular marker of the Type II CRISPR-Cas system. However, it is unknown via which cellular pathways CjeCas9 drives human intestinal epithelial cells into cell death. Here, we show that CjeCas9 released by *C. jejuni* during the infection of Caco-2 human intestinal epithelial cells directly modulates Caco-2 transcriptomes during the first four hours of infection. Specifically, our results reveal that CjeCas9 activates DNA damage (p53, ATM (Ataxia Telangiectasia Mutated Protein)), pro-inflammatory (NF-κB (Nuclear factor-κB)) signaling and cell death pathways, driving Caco-2 cells infected by wild-type *C*. *jejuni*, but not when infected by a *cas9* deletion mutant, towards programmed cell death. This work corroborates our previous finding that CjeCas9 is cytotoxic and highlights on a RNA level the basal cellular pathways that are modulated.

## 1. Introduction

*Campylobacter jejuni* is a zoonotic bacterial pathogen that causes gastrointestinal infections, with symptoms including (bloody) diarrhea or dysentery-like conditions such as cramps, fever and pain [1], also known as Campylobacteriosis. Clinical manifestations of *C*. *jejuni* infections are the consequences of the ability of pathogenic *C. jejuni* bacteria to disrupt the intestinal epithelial barrier [2,3], concomitant with DNA damage and intestinal epithelial cell death [4,5]. In this process, there are important roles for virulence factors that mediate adhesion onto, invasion into and translocation across intestinal epithelial cells, and bacterial motility [6,7,8,9,10]. In vitro, we and others have shown that *C*. *jejuni* adheres onto and invades into intestinal epithelial cells in a two to four hour time window, in which sialylated lipo-oligosaccharides (LOS), enterotoxins, cytotoxins, adhesins and motile flagella play important roles [5,6,7,8,9,10,11,12]. Upon epithelial cell infection, *C*. *jejuni* co-localizes with cellular endosomal markers at specific time points [13,14]. This 2–4 h process is a crucial time period, since it determines if *C*. *jejuni* are killed in the phagolysosome or if bacteria survive and translocate across the intestinal epithelial barrier [12,13].

To infect and damage host cells, some bacteria target host DNA [15]. In *C*. *jejuni*, Cytolethal Distending Toxin (CDT) can damage host DNA [15,16,17,18], but isolates lacking CDT still induce DNA damage, cell death [4] and campylobacteriosis [19,20], indicating that additional virulence factors contribute to host DNA damage. We revealed that the protein encoded by Clustered Regularly Interspaced Palindromic Repeat and associated gene 9 (*cas9*) of *C*. *jejuni* (CjeCas9) is necessary to induce DNA damage and cell death during the infection process in a variety of cell lines [14]. In *C. jejuni*-infected cells, the DNA damage markers 53BP1 and y-H2AX are massively activated and contribute to the repair of CjeCas9 related DNA damage [14]. Others have shown that *C*. *jejuni* can translocate across human intestinal epithelial barrier in the presence of CjeCas9 [21], and that translocation is accompanied with a significant drop in the transepithelial electrical resistance (TER) [12,22]. The most parsimonious explanation for these findings is that CjeCas9 might directly induce cytotoxicity via one or multiple processes. In order to pinpoint these processes, we carried out infection assays with Caco-2 intestinal epithelial cells using a wild-type strain and a corresponding isogenic *cas9* deletion mutant [21] and monitored the infection process by extracting total RNA at biologically relevant time points that we earlier determined [11,12] for whole-genome transcriptomics. We intended to validate our hypothesis that CjeCas9 activates the DNA damage and cell fate pathways [14,21].

## 2. Materials and Methods

### 2.1. Bacterial Strains and Growth Conditions

*Campylobacter jejuni* strain NCTC11168 with a complete CRISPR-Cas system is used in this study [12,21,23]. The methods for generating a Δ*cas9* mutant strain have been described previously [12,21]. *C. jejuni* was cultured on blood agar plates, containing 7% sheep blood (Becton Dickinson, Breda, the Netherlands) supplemented with vancomycin (10 µg/mL). The isogenic Δ*cas9* mutant, generated in the NCTC11168 background, was cultured using the above medium supplemented with chloramphenicol (20 µg/mL) (Sigma-Aldrich, Zwijndrecht, the Netherlands). *C. jejuni* was cultured under micro-aerophilic conditions at 37 °C, using anaerobic jars and an Anoxomat (Mart Microbiology B.V., Drachten, the Netherlands).

### 2.2. Eukaryotic Cell Maintenance

The human intestinal epithelial cell line Caco-2 (human epithelial colorectal adenocarcinoma cells) was maintained in Dulbecco’s modified Eagle’s medium (DMEM) (Thermo Fisher Scientific, Bleiswijk, the Netherlands) supplemented with 10% fetal bovine serum (FBS) (Thermo Fisher Scientific, Bleiswijk, the Netherlands), 100 U/mL penicillin, 100 µg/mL streptomycin and 1% non-essential amino acids (NEAAs) (Thermo Fisher Scientific, Bleiswijk, the Netherlands). The Caco-2 cells were cultured in a 75-cm^2^ flask (Greiner Bio-one, Alphen aan den Rijn, the Netherlands) at 37 °C and 5% CO_2_ in a humidified air incubator.

### 2.3. Transwell Cellular Assay

Transwell filters (Costar, Corning Inc., Corning, New York, NY, USA) were coated with collagen (50 μg/mL in 0.02 M acetic acid). Caco-2 cells were seeded at the apical surface of coated Transwell filters at a density of 4.0 × 10^5^ cells/filter (5-μm pore size, 1.13 cm^2^; (Costar) in 400 μL DMEM medium (Thermo fisher scientific) + Glutamax (Thermo fisher Scientific, Bleiswijk, the Netherlands) with 10% FBS (Thermo fisher Scientific), 1% glutamine (Thermo fisher Scientific, Bleiswijk, the Netherlands) and 1× Penicillin/Streptomycin (Thermo fisher Scientific). Nine hundred microliter of the above medium was added to the basolateral surface and 400 microliter was added to the apical surface of the Caco-2 cells, which were allowed to differentiate for 19–21 days with medium replacements every other day accompanied with transepithelial electrical resistance (TER) measurements. Plates were incubated at 37 °C in 5% CO_2_ in a humidified incubator (Binder, Tuttlingen, Germany). TER above >1000 Ω/cm^2^ indicated that intact epithelial monolayers were present, usually at day 19 [12] and can be used as a model for the human epithelial barrier [24].

When carrying out infection assays, the medium described above was replaced with the same medium, but lacking antibiotics. Bacterial strains were added at a multiplicity of infection (MOI) of 10 to the apical surface of the Caco-2 cells at day 19–21. After 48 h, Transwells (Costar) were rinsed with phosphate-buffered saline (PBS) (Thermo Fisher Scientific, Bleiswijk, the Netherlands) at 37 °C and fixed with 4% paraformaldehyde (Sigma-Aldrich, Zwijndrecht, the Netherlands) for one hour. Transwells were washed in PBS and dehydrated in 70% ethanol (Sigma-Aldrich, Zwijndrecht, the Netherlands) (2× 15 min), 96% ethanol (2× 20 min), 100% ethanol (1× 10 min and 2× 20 min) and 100% butanol (Sigma-Aldrich, Zwijndrecht, the Netherlands) (1× 20 min and 2× 30 min). Membranes were embedded in paraffin (Sigma-Aldrich, Zwijndrecht, the Netherlands) and stored at room temperature until sectioned. Then, 5-μM-thick slides were deparaffinated in xylene (Sigma-Aldrich, Zwijndrecht, the Netherlands), dehydrated in a graded ethanol series (100%, 96%, 90%, 80%, 70%, 50%) and finally rinsed in H_2_O. Transwell sections were stained with Hematoxylin (Sigma-Aldrich, Zwijndrecht, the Netherlands) and Eosin (HE staining) (Sigma-Aldrich, Zwijndrecht, the Netherlands) and analyzed using the microscope at 40× magnification using bright-field illumination.

### 2.4. RNA and Microarray Handling for Transcriptomics of C. jejuni Infected Human Cells

To investigate genome-wide transcriptional responses of Caco-2 intestinal epithelial cells to infection with *C. jejuni*, Caco-2 cells were seeded in 6-well plates (Greiner Bio-One) at a density of 1.0 x 10^5^ cells per well, grown to confluence and allowed to differentiate for 19 days and infected. Three biological replicates (3 different wells) were used per wild-type (WT) and its isogenic Δ*cas9* mutant per time point. At the t = 0 hr time point, cells were incubated with bacteria or medium only (mock infection challenge). Five additional time points of RNA extraction—30, 60, 120, 180, and 240 min—after infection were rationally chosen based on our earlier work [12]. At each time point, 1 mL of TRIzol^®^ reagent (Ambion Life Technologies) was added to the appropriate wells and total RNA was extracted from the Caco-2 cells following the manufacturer’s protocol. Air-dried RNA was re-suspended in 100 µL MilliQ water and purified and desalted using Qiagen RNeasy Mini Kit spin columns following the manufacturer’s instructions. RNA quantity and quality was assessed spectrophotometrically via a Nanodrop device (ND-1000, NanoDrop Technologies, Wilmington, DE, USA) and with 6000 Nano chips via a Bioanalyzer 2100 device (Agilent, Santa Clara, CA, USA), respectively. RNA was judged as being suitable for array hybridization only if samples showed intact bands corresponding to the 18S and 28S ribosomal RNA subunits, displayed no chromosomal peaks or RNA degradation products, and had a RIN (RNA integrity number) above 8.0. The Ambion WT Expression kit (Life Technologies, cat. no. 4411974) in conjunction with the Affymetrix GeneChip WT Terminal Labelling kit (Affymetrix, Santa Clara, CA, USA; cat. no. 900671) was used for the preparation of labelled cDNA from 100 ng of total RNA without rRNA reduction. Labelled samples were hybridized on Affymetrix GeneChip Human Gene 1.1 ST arrays that contain 30,000 coding transcripts and over 11,000 long intergenic non-coding transcripts, provided in plate format. The hybridization, washing and scanning of the array plates were performed on an Affymetrix GeneTitan Instrument, according to the manufacturer’s recommendations. Detailed protocols can be found in the Affymetrix WT Terminal Labelling and Hybridization User Manual (part no. 702808 revision 4), and are also available upon request. Quality control of the hybridizations to the Human Gene 1.1 ST array and primary data analysis were performed according to strict criteria to ensure that the array data were of the highest possible quality (below).

### 2.5. Statistical and Functional Analysis of Microarray Data

Packages from the Bioconductor project [25] were used for analyzing scanned Affymetrix transcriptome arrays. Arrays were normalized using quantile normalization, and expression estimates were compiled using the pre-processing algorithm Robust Multi-array Analysis (RMA), applying the empirical Bayes approach available in the Bioconductor library affyPLM using default settings. Gene functional annotations, gene ontology (GO) enrichment and differential expression calculations were carried out using Bioconductor [25] packages and third-party software modules (see below). The Bioconductor packages were integrated in the automated on-line MADMAX pipeline [26]. Various advanced quality metrics, diagnostic plots, pseudo-images and classification methods were applied to ascertain that only arrays that passed the most rigorous quality controls were used in the subsequent analyses [27]. Arrays were considered of sufficient quality when they showed no more than 10% of specks in fitPLM model images, were not deviating in RNA degradation and density plots, when they were not significantly deviating in Normalized Unscaled Standard Error (NUSE) and Relative log expression (RLE) plots and were within each other’s range in boxplots. For a more extensive description of quality criteria, please contact the authors. Probe sets were redefined according to Dai et al. [28] utilizing current genome information. In this study, probes were reorganized based on the NCBI (National Center for Biotechnology Information) Entrez Gene database (remapped CDF v15, http://brainarray.mbni.med.umich.edu). Differentially expressed probe sets were identified using linear models, applying moderated t-statistics that implement empirical Bayes regularization of standard errors using Bioconductor’s limma package [29]. A Bayesian hierarchical model was used to define an intensity-based moderated T-statistic (IBMT), which takes into account the degree of independence of variances relative to the degree of identity and the relationship between variance and signal intensity [30]. When gene expression was low and just below significance, results of the limma test were compared to the IBMT test. *p*-values were corrected for multiple testing using a false discovery rate (FDR) method [31]; the quality of the data was such that FDR *p*-values (Q values) of <0.01 to <0.0001 yielded hundreds of, to several thousand, differentials, depending on the time point and *C. jejuni* strain. For pathway analysis comparisons, it is difficult to use different numbers of input genes (several hundred compared to several thousand), which would occur if a fixed FDR *p*-value was used for all datasets submitted to pathway analysis. Therefore, FDR values between *p* < 0.01 and *p* < 0.0001 were chosen such that the number of genes included in Ingenuity Pathway Analysis (IPA) and Cytoscape [32] were in the range of 700–1000 genes.

### 2.6. Biological Interpretation of Transcriptome Datasets

Time-resolved differential gene expression data were obtained using Short Time-series Expression Miner (STEM) software [33]. To identify pathways and processes among regulated genes activated in response to infection by NCTC11168 or its isogenic Δ*cas9* mutant at each time point, Ingenuity Pathways Analysis (IPA) (Ingenuity Systems, Redwood City, CA, USA) was used (below). For network analysis, the same infection response datasets were imported into Cytoscape [32] and interactions of the proteins encoded by the differentials were obtained via the Bisogenet plugin. The output was used to prioritize differentially regulated pathways that reflect Caco-2 responses to bacterial infection, to identify cascades of upstream transcriptional regulators that could explain the observed gene expression changes, and to reconstruct protein–protein networks that could be used to overlay gene expression data and identify central regulatory proteins that were most likely to have driven differential gene expression following infection by *C. jejuni*. We used three complementary methods for functional analysis of microarray expression data: ErmineJ (GO annotation enrichment or overrepresentation), gene set enrichment analysis and IPA (see below). Using these, we performed: (i) identification of statistically supported overrepresentation of functional gene ontology (GO) annotation, (ii) mapping of expression data onto pathways to determine their up- or downregulation in a statistical meaningful way (IPA), (iii) projection of transcript fold-change values of co-expressed genes onto interaction maps of the corresponding proteins (IPA and Cytoscape), and (iv) the reconstruction of networks from the interactions of proteins expressed by all gene differentials per dataset to identify central regulators. Two complementary methods were applied to relate changes in gene expression to functional changes. One method, ErmineJ, is based on the overrepresentation of Gene Ontology (GO) terms [34]. As input all t-test *p*-values from the probe set comparisons across the respective conditions were used. Another approach, gene set enrichment analysis (GSEA) takes into account the broader context in which gene products function, namely in physically interacting networks, such as biochemical, metabolic or signal transduction routes [35]. This method aids the identification of up- or downregulated processes. Due to overlap in the source databases, cellular functions may be represented multiple times. Both applied methods have the advantage that they are unbiased, because no gene selection step is used, and a score is computed based on all genes in a particular GO term or gene set. To evaluate the statistical support for similarity of expression datasets as calculated by GSEA, we made use of the three *p*-values provided by the GSEA algorithm. Statistical support was considered sufficiently convincing when meeting the following criteria: nominal *p*-values < 0.05, FDR *p*-values < 0.2, and FWER values < 0.25. In practice, GSEA conclusions were based on *p*-values closely approaching 0.

### 2.7. Pathway Analysis

All listed or reconstructed cellular pathways were derived from the expert-annotated pathways that are provided by the Kyoto Encyclopedia of Genes and Genomes (KEGG www.genome.jp/kegg) and the Ingenuity Knowledge Base (www.ingenuity.com) or were reconstructed from known protein–protein and protein–DNA interaction information that is present in on-line databases including those from NCBI, Ensembl, BIND (Biomolecular Interaction Network Database), KEGG and MIPS (Munich Information Center for Protein Sequences). Furthermore, the localization and expression of the proteins that were encoded by differentially expressed genes were cross-checked in the Human Protein Resource Database (www.hprd.org). This resource not only provides information on tissue-specific expression of human proteins, but also includes histological images showing cell-specific expression for many of the proteins discussed in this manuscript. As a consequence, the data interpretation was exclusively built on existing and curated information, and all interpretations were based on validated information. Biological cellular functions and transcriptional networks altered after the consumption of bacterial preparations were identified using IPA; Ingenuity Systems, Redwood City, CA. IPA annotations follow the GO annotation principle, but are based on a proprietary knowledge base of over 1,000,000 protein–protein interactions. For IPA analysis, gene expression ratios x between 0 and 1 were transformed to negative fold-changes using the formula fc = −1/(ratio x). We also performed an IPA upstream regulator analysis to identify the cascade of upstream transcriptional regulators that can explain the observed gene expression changes in a user’s dataset, which can provide insights into the biological activities occurring in the tissues or cells being studied. The IPA output includes metabolic and signaling pathways with statistical assessment of the significance of their representation being based on Fisher’s Exact Test. This test calculates the probability that genes participate in a given pathway relative to their occurrence in all other pathway annotations. Input gene lists included the differentially regulated genes (FDR *p*-values (Q values) < 0.001 or <0.0001 where appropriate; we aimed to include 700–1000 genes for IPA analysis, since the statistical output of the Fisher’s Exact Test that IPA uses is sensitive to highly diverse numbers of input genes. IPA computes networks and ranks these according to a statistical likelihood approach. All networks with a score of at least 10 focus genes were considered to be biologically relevant and representative to show part of the underlying biology of the responses of Caco-2 cells to infection challenges by WT or its isogenic Δ*cas9* mutant. Every interaction between gene products in the network was supported by published information that was directly retrieved from within the Ingenuity knowledge base. In order to identify major activated networks and pathways, a fold-change cut-off value of two was used. In order to reconstruct cellular pathways including those genes that showed significantly altered expression, a fold-change cut-off of 1.1 was used. Small (10–40%) changes in gene expression in human tissue induced by mild stimuli have been published earlier [36] and may be a characteristic of mean expression changes in human tissue, typically containing cellular regions with possibly differing gene expression programs. Common (canonical) pathway results obtained using software suites such as IPA are partially determined by the content of the supporting databases, the “knowledge base”. To further evaluate the IPA results, we used a second type of in silico pathway analysis, delivered by the Cytoscape platform that uses all main publicly available databases including STRING, NCBI, Uniprot and KEGG, in contrast to Qiagen IPA, which is based on a proprietary manually curated data of human, mouse and rat protein–protein, protein–DNA and protein–compound interactions. To access these public databases, the Bisogenet, PSICQUICUniversalClient and StringWSClient plugins were used to construct protein–protein interaction networks representing the proteins encoded by the differentially expressed genes. Major nodes, candidate regulators of the Caco-2 response to infection with WT or isogenic Δ*cas9* mutant bacteria, were identified using the cytoHubba plugin. All plugins and the documentation describing them can be retrieved via http://apps.cytoscape.org.

## 3. Results

### 3.1. CjeCas9 Triggers Caco-2 Cell Damage

**Figure 1:**
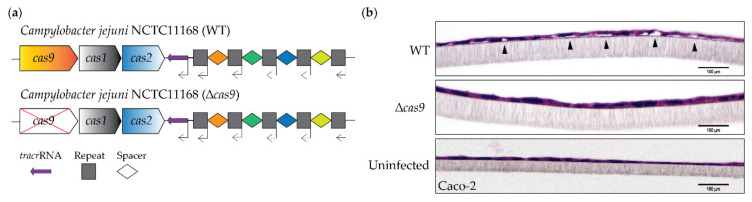
CjeCas9 induces cell damage. (**a**) Organization of the *C. jejuni* CRISPR-Cas locus in the wild-type NCTC11168 (WT) strain and its isogenic *cas9* deletion mutant (∆*cas9*). Transcriptional direction of the *cas* genes are indicated [38]; (**b**) Transwell sections showing CjeCas9-induced swelling in differentiated Caco-2 cells at 48 hpi. Transwell sections were fixed and stained with HE (Hematoxylin and Eosin). Images were taken by phase contrast microscope; scale bars represent 100 µm.

CjeCas9 is required for the efficient *C*. *jejuni* infection of human cells including cell death induction [21,37]. To investigate the contribution of CjeCas9 to the induction of host damage and death, we used the NCTC11168 (WT) strain and its isogenic ∆*cas9* mutant (Figure 1a) in epithelial cell (Caco-2) infection assays. Challenges with the wild-type CjeCas9-producing strain resulted in the induction of swelling of differentiated human intestinal epithelial Caco-2 cells, indicated by arrows in the upper panel of (Figure 1b). Such cell swelling was not observed when these Caco-2 cells were exposed to the isogenic ∆*cas9* mutant (Figure 1b).

### 3.2. CjeCas9 Modulates Transcriptomes of Caco-2 Cells during the Early Stages of Infection

To gain insight into the molecular basis behind Caco-2-induced cell damage by CjeCas9-producing *C. jejuni* [14], we analyzed the Caco-2 transcriptome at five rationally chosen infection time points obtained after infection. The raw data are publicly available at NCBI Gene Expression Omnibus (GEO), accession series GSE89661. Extensive tables including GO enrichment and other analysis results can be requested from the authors; the most relevant results are summarized here.

To get a first impression of significantly altered gene expression across all transcriptome samples, non-parametric Kruskal–Wallis and Rank Products (RP) tests [39] were used. The heatmap output of these tests showed that Caco-2 genes involved in basal and DNA metabolism and cell cycle were consistently differentially regulated across the arrays in response to bacterial infection, with a clear distinction in earlier and later infection time points and between WT and its isogenic ∆*cas9* mutant (Figure S1). These results hint at which major processes had been altered in Caco-2 cells during the first four hours when challenged with WT or its isogenic ∆*cas9* mutant but are not sensitive enough to identify which pathways had been differentially regulated across time.

### 3.3. Challenges with WT C. jejuni Strains Alter Gene Expression Profiles of Caco-2 Cells over Time and Are Associated with Cell Damage

**Figure 2:**
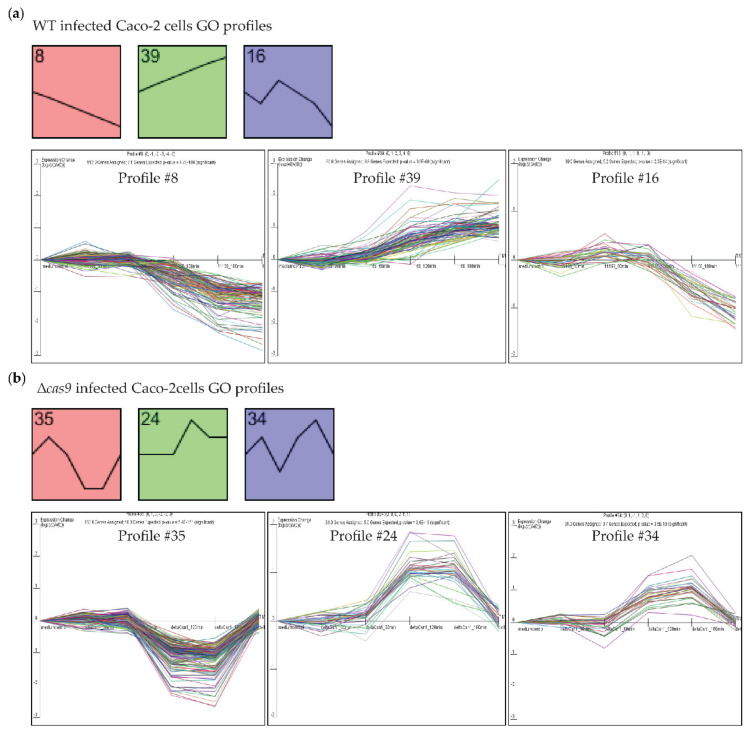
CjeCas9 induces cellular damage by modulating gene expression in Caco-2 cells. (**a**) The Table S2 cell transcriptomes after infection with the wild-type CjeCas9-producing *C. jejuni* strain (WT) could be clustered in three profiles of genes that were (i) significantly differentially expressed across time and that (ii) were enriched for specific Gene Ontology (GO) terms (*p*-values: Profile 8 = 4.7^−100^, Profile 39 = 3.6^−60^ and Profile 16 = 2.6^−14^). The line graphs show the trend of gene expression; individual lines represent individual genes. (**b**) Time-resolved Caco-2 cell transcriptomes after infection challenge with the isogenic ∆*cas9* mutant could be clustered in three profiles of genes that were significantly differentially expressed across time and enriched for specific GO terms (*p*-values: Profile 35 = 5.4^−151^, Profile 24 = 3.6^−18^ and Profile 34 = 3.5^−19^).

To identify Caco-2 genes with differential expression profiles across all five time points following challenge by WT or isogenic *cas9* deletion mutant strain, time series analysis using Short Time Series Expression Miner (STEM) software (see the methods section) was used to obtain significantly differentially expressed, time-resolved Caco-2 gene expression profiles. STEM analysis clusters genes based on similar gene expression across the (five) time points by comparing expression profiles to pre-modelled trends (“Profiles”) (for example, cluster genes all being consistently downregulated across the five time points as in Profile 8 below), and by calculating GO enrichment for each gene cluster. Three STEM profiles were significantly enriched for specific Gene Ontology (GO) terms after infection by WT (Figure 2a and Table S1). Profile 8 included genes that had been consistently downregulated during infection of Caco-2 cells with WT. Profile 8 genes were enriched for GO terms representing DNA packaging, nucleosome assembly and organization, and DNA binding and gene expression, suggesting that these processes had been suppressed during infection by WT. Profile 39 included genes that had been consistently upregulated during WT infection of Caco-2 cells. Profile 39 genes were enriched for GO terms representing response to wounding, hypoxia, negative regulation of cell adhesion and cell migration, suggesting that these processes had been induced during infection by WT *C. jejuni*. Profile 16 included genes that had been induced during earlier time points and downregulated at later time points during WT infection. Profile 16 genes were enriched for GO terms representing kinase activity, cell cycle and cell proliferation, suggesting that the regulation and suppression of the Caco-2 cell cycle had been modulated via kinase activity. STEM analysis of Caco-2 cells challenged with the isogenic ∆*cas9* mutant yielded three profiles of co-expressed human genes that were enriched for specific GO terms (Figure 2b and Table S1). Profile 35 included genes that had been downregulated at earlier time points upon which their expression increased to “t = 0” at later time points. Profile 35 genes were enriched for GO terms representing DNA packaging, nucleosome assembly and organization and DNA binding. Profile 24 and Profile 34 included genes that were upregulated at earlier time points upon which their expression decreased to “t = 0” at later time points. Profile 24 and Profile 34 genes were enriched for GO terms representing epithelial cell differentiation, adhesion, proliferation and development, apoptosis and protein metabolic processes including phosphorylation.

### 3.4. Challenging Caco-2 Cells with a Wild-Type CjeCas9-Producing C. jejuni Strain is Associated with the Induction of Cell Death and Pro-Inflammatory Signaling Pathways

**Figure 3:**
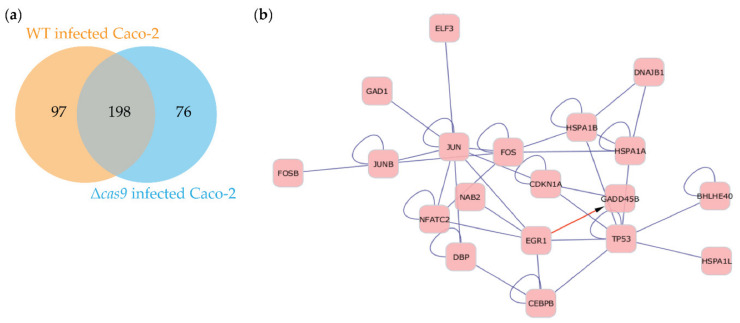
198 genes are shared between time-resolved transcriptomes of Caco-2 cells challenged by wild-type CjeCas9-producing *C. jejuni* strain (WT) or its isogenic ∆*cas9* mutant (**a**) STEM analysis of Caco-2 cells transcriptomes infected by WT and its isogenic ∆*cas9* mutant yielded a set of 198 genes that were significantly modulated upon infection by either strain. (**b**) Cytoscape network analysis (see the methods section) revealed that the proteins encoded by these genes formed a network including the cell cycle regulators JUN, FOS and p53 (encoded by the gene TP53) as central nodes.

Based on our earlier work [14], it was of interest to us to identify candidate regulators of the cell death and pro-inflammatory pathways that were continuously induced by the wild-type CjeCas9-producing *C. jejuni* strain. Interestingly, one set of 198 Caco-2 genes was regulated across time by both WT and its isogenic ∆*cas9* mutant (Figure 3a and Table S2). These genes were enriched for GO terms associated with DNA metabolism, gene expression, cell fate and cell cycle regulation. Cytoscape network analysis (see the methods section) revealed that the proteins encoded by these 198 genes formed a network that included the cell cycle and immune response regulators JUN (jun proto-oncogene), FOS (Finkel-Biskis-Jinkins osteosarcoma) and p53 (encoded by the gene TP53) as central nodes (Figure 3b). p53, a major stress protein, co-occurred in the network with the GADD45B protein, that is encoded by the growth arrest and DNA-damage-inducible-alpha gene that is induced by DNA-damaging agents. This was of interest, since two non-parametric tests showed that genes involved in DNA and chromatin metabolism had been significantly differentially expressed across all the individual Caco-2 transcriptome of group 1 and 2 (Figure S1). We therefore further explored the 198-gene set using global gene ontology and functional annotations and pathway mapping using the online tool DAVID [40].

The genes included in the 198-gene set were enriched in pathways regulating apoptosis (PANTHER), MAPK (Mitogen-activated protein kinase) signaling (KEGG), IL-6 signaling, hypoxia and ATM signaling via p53 (DNA damage) pathways (BioCarta); pathway databases in parentheses are included in DAVID (data not shown). This was of interest since *C. jejuni* drives IL-6 secretion [41], MAPK and hypoxia signaling and apoptosis, corroborating DAVID functional predictions for the 198-gene set. Furthermore, about half of the significantly expressed genes found in the non-parametric tests across all samples were involved in DNA and chromatin metabolism (Figure S1).

**Figure 4:**
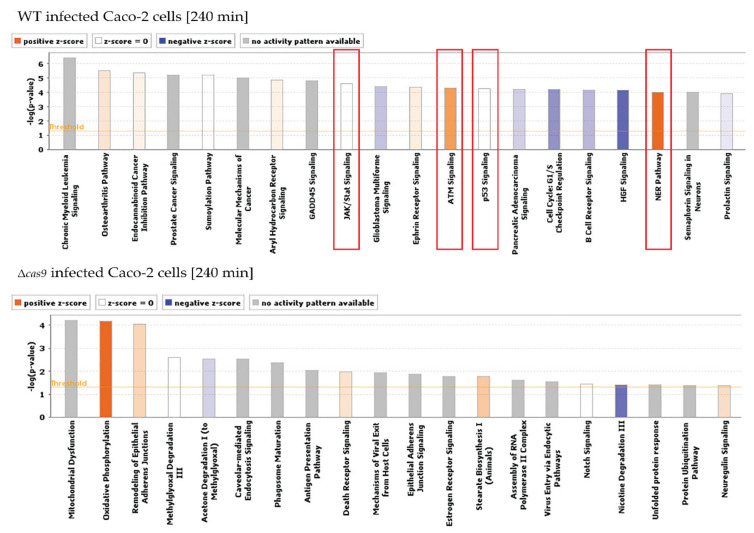
Cellular pathways significantly modulated, induced or repressed in Caco-2 cells at early time points of infection by WT or its isogenic *Δcas9* mutant. Pathway analysis was carried out using Ingenuity Pathway Analysis (IPA). Infection by wild-type *C. jejuni* leads to a significant modulation of the p53 and JAK-STAT pathways and an induction of DNA-damage response ATM signaling and Nucleotide-Excision Repair (NER) pathways (red boxes). These stress-associated pathways were not significantly modulated during infection by the isogenic *∆cas9* mutant (lower graph). In these graphs, the horizontal yellow line indicated as “Threshold” represents –log (*p*-value = 0.05); bars higher than that line represent significantly modulated pathways and processes.

The network analysis suggested that JUN, FOS and p53 might be central regulators in the Caco-2 cells when challenged by *C. jejuni*, specifically by CjeCas9-producing *C. jejuni*. This was of interest to us since our previous work suggested that continuous exposure to CjeCas9 is followed by cell death [14]. To investigate the notion that infection by Cas9-producing *C. jejuni* induces cellular stress via p53 after four hours, but not during infection by Cas9-non-producing *C. jejuni*, gene expression was compared for Caco-2 cells challenged by WT or its isogenic ∆*cas9* mutant via the limma t-test (see the methods section). Caco-2 genes that were significantly differentially expressed at four hours post-infection were used as input in Ingenuity Pathway Analysis (IPA), which revealed that the WT isolate was significantly able to induce pathways in Caco-2 cells that are involved in stress responses including DNA damage (Figure 4 and Table S3), thus in line with our earlier findings [14]. However, these pathways were not induced during infection challenges of Caco-2 cells by the isogenic ∆*cas9* mutant, showing that loss of Cas9 results in the inability of *C. jejuni* to induce stress- and DNA damage-signaling pathways in Caco-2 cells, whereas the corresponding WT strain can do so (Figure 4 and Table S3). Moreover, exploration of significantly modulated pathways after the infection of Caco-2 cells by WT showed that, together with p53, NF-κB signaling was induced including the pathway genes TNFR, A20, caspase-8, p65(RelA)NF-κB, and CPB/p300, which modulate cell death and pro-inflammatory signaling (Figures S2 and S3). In contrast, the p53 and NF-κB signaling pathway in Caco-2 cells were not significantly modulated at four hours post-infection by the isogenic ∆*cas9* mutant (Figures S2 and S3; Table S3).

## 4. Discussion

The enteric zoonotic human pathogen *C. jejuni* may translocate across intestinal epithelial cells [14,15,21]. Depending on a strain’s genotype and production of virulence factors, this process may be associated with cell death, but characterization of toxins has been challenging [5,7]. In our previous work, we found that, during infection, pathogenic *C. jejuni* bacteria secrete CjeCas9 via outer membrane vesicles in the cytoplasm of human cells. After release into the cytoplasm, a native nuclear localization signal mediates entry of CjeCas9 into the nucleus, where it targets the DNA [14]. The observed swelling of the Caco-2 cells might therefore be a direct effect of the severity of DNA damage induced by CjeCas9 in the nucleus, affecting homeostasis of the infected cell, more specifically, homeostasis of ions and water in the cytosol.

Earlier, we have shown that the CjeCas9 nuclease plays a key role in the *C*. *jejuni* infection process [11,12,21], and that CjeCas9 causes severe damage to the human genome in the presence of metal ions magnesium and manganese [14,21]. Metal ions used by this endonuclease are enriched in the jejunum, the preferred site of infection in the human intestine by *C*. *jejuni* [42,43,44,45]. In the current work, we extended our findings by revealing which human pathways and processes are modulated by CjeCas9 in intestinal epithelial cells. We used polarized Caco-2 cell monolayers since when these epithelial cells are grown as confluent monolayers on micro-porous membranes, they produce the typical microvilli and a well-defined brush borders that are characteristic for human intestinal epithelia [46]. We found that the Caco-2 cell response pathways modulated by a CjeCas9-producing strain include DNA damage, cell death and pro-inflammatory pathways. Our findings are in line with the DNA damaging and cell death pathology that is specifically observed during the *C*. *jejuni* infection process [14]. The pro-inflammatory pathways modulated by this bacterium might be relevant in chronic inflammation of the human intestine, one of the post-infectious complications associated with *C. jejuni* infections [47]. In fact, *C*. *jejuni*-induced tissue damage is a well-known pathogenic feature demonstrated in biopsies obtained from the intestine of infected patients and in in vitro assays [48,49] including the swelling of the intestinal epithelial barrier that we also observed in the present work—a finding reproducible with other WT strains and their isogenic ∆*cas9* mutants (results not shown). Our present and earlier work has shown the contribution of CjeCas9 to this *C. jejuni* pathology [14,21], supporting the notion that CjeCas9 functions as a virulence factor.

In previous work, we demonstrated that, six hours post-infection, infected cells exposed to CjeCas9 accumulated p53-binding protein 1 (53BP1) and the phosphorylated histone H2A variant X (γ-H2AX) into their nuclei [14]. 53BP1 and γ-H2AX both play key roles in the DNA damage response [14] and interact with p53 and the corresponding signaling pathway that regulates cell cycle, DNA repair, genome stability and programmed cell death [50,51,52,53]. Modulation of the p53 signaling pathway by Cas9 nucleases may be a more general feature. For example, the Cas9 protein of the bacterial pathogen *Streptococcus pyogenes*, SpyCas9, activates the p53 pathway in a wide variety of human cell lines [54,55,56].

Another host factor commonly associated with *C. jejuni* infection is NF-κB, which regulates cell survival genes together with genes involved in the inflammatory response [57,58] and the DNA damage response [59,60]. NF-κB activation by *C*. *jejuni* is reported to occur independently of TLR-2, TLR-4, Nod1 and Nod2 receptors and suggests a novel mechanism [58]. In gnotobiotic IL-10^−/−^; NF-κB(EGFP) mice, NF-κB induction in lamina propria mononuclear cells was associated with ulcerating colonic inflammation and bloody diarrhea [61]. Our study now shows that the CjeCas9 protein contributes to NF-κB activation during successful *C. jejuni* infections.

During the first two-three hours of infection by WT or its isogenic ∆*cas9* mutant similar cell fate-associated genes are modulated in Caco-2 cells. However, the continuous induction of cell-death-promoting genes occurred only in Caco-2 cells during infection by wild-type CjeCas9 producing *C. jejuni*, whereas, during infection with the isogenic ∆*cas9* mutant, the expression of cell-death-promoting genes normalized after 2–3 h to levels measured for mock-challenged Caco-2 cells. Our work thus suggests that CjeCas9 is a key modulator of the DNA damage and cell death response pathways that are induced upon infection of human intestinal cells by *C*. *jejuni* [14]. In conclusion, this work corroborates our previous finding that CjeCas9 is cytotoxic and highlights, at the RNA level, the basal cellular pathways that are modulated in Caco-2 cells when exposed to CjeCas9.

## Supplementary Materials

The following are available online at https://www.mdpi.com/2073-4425/11/10/1193/s1, Figure S1: CjeCas9 modulates the Caco-2 transcriptome during infection, Figure S2: IPA shows that wild-type CjeCas9-producing *C. jejuni* strain (WT), but not its isogenic ∆*cas9* mutant modulates the p53 signaling pathway during infection of Caco-2 cells, Figure S3: IPA shows that wild-type CjeCas9-producing *C. jejuni* strain (WT), but not its isogenic ∆*cas9* mutant modulates the NF-κB signaling pathway during infection of Caco-2 cells, Table S1: Gene Ontology (GO) profiles from STEM analysis, Table S2: List of differentially expressed genes, Table S3: Significantly modulated canonical pathways (Ingenuity Pathway Analysis output) after infection of Caco-2 cells by WT *C. jejuni* or its isogenic ∆*cas9* mutant.
