# Supplementary material for: Campylobacter jejuni Cas9 Modulates the Transcriptome in Caco-2 Intestinal Epithelial Cells
Source: Genes (Basel). 2020 Oct 14;11(10):1193. doi: 10.3390/genes11101193 (PMC7650535; doi:10.3390/genes11101193)
Supplement: Supplementary file 1 [file genes-11-01193-s001.zip › Supplementary Files/Supplementary figures.docx]

**Supplementary figures**


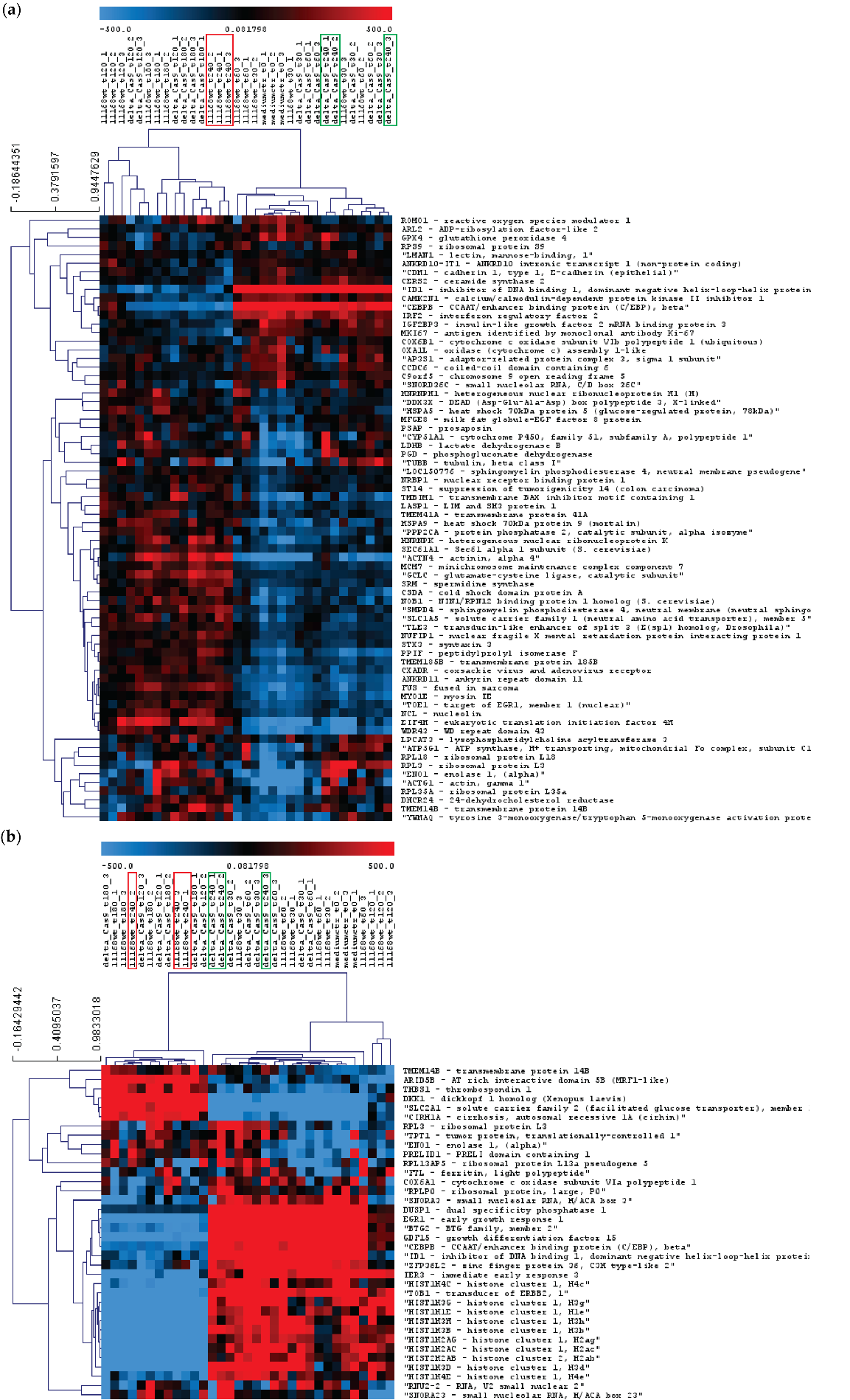


**Figure S1.** CjeCas9 modulates the Caco-2 transcriptome during infection. (a and b) Transcriptome analysis of Caco-2 cells infected by the WT strain (11168wt) and its isogenic ∆*cas9* mutant (delta_cas9). (a) Non-parametric Kruskal-Wallis ANOVA (p < 0.05) and (b) Rank products test (RP) identified significantly regulated genes across all samples. For RP test a two-class design was used, with WT infected Caco-2 cells as group 1 input genes and its isogenic ∆*cas9* mutant infected Caco-2 cells as group 2 input genes. Four hours post-infection time point samples are indicated for WT or its isogenic ∆*cas9* mutant by red boxes and green boxes, respectively.


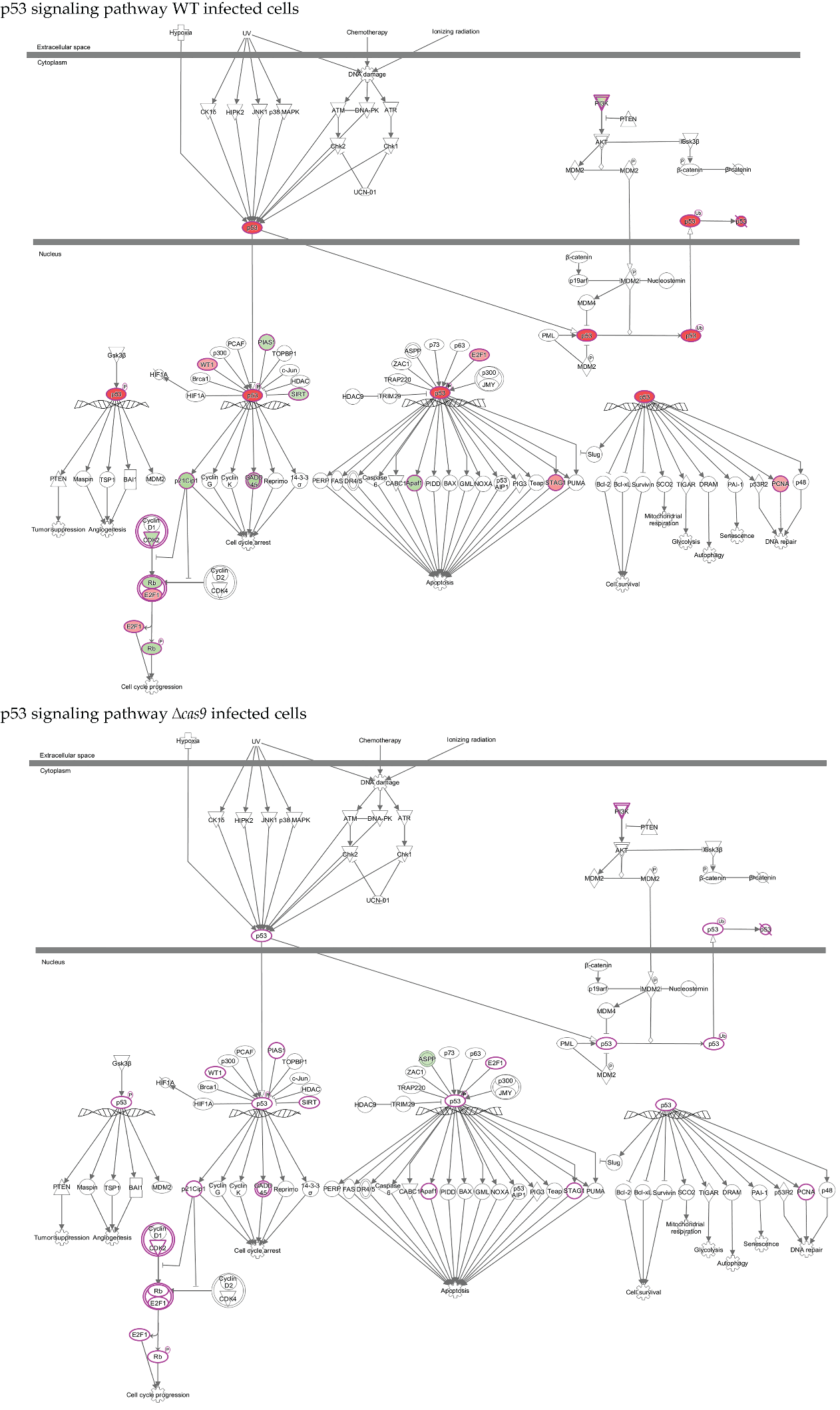


**Figure S2.** IPA shows which Caco-2 p53 signaling pathway genes were significantly modulated upon infection by wild-type Cas9-producing *C. jejuni* strain (WT; upper panel), and by its isogenic *cas9* deletion mutant (∆*cas9*; lower panel). During infection by WT, the gene encoding p53 stress protein was upregulated and crucial cell cycle progression genes including Rb (retinoblastoma) and CDK2 were downregulated; the p53 signalling pathway had been significantly modulated (Figure 4). These genes were not differentially expressed during infection by the isogenic ∆*cas9* mutant and the p53 signaling pathway was not significantly modulated during infection by this mutant (Figure 4). In the pathway maps, red color represents upregulation of gene expression and green color represents downregulation of gene expression; expression levels are relative to mock-challenged cells.


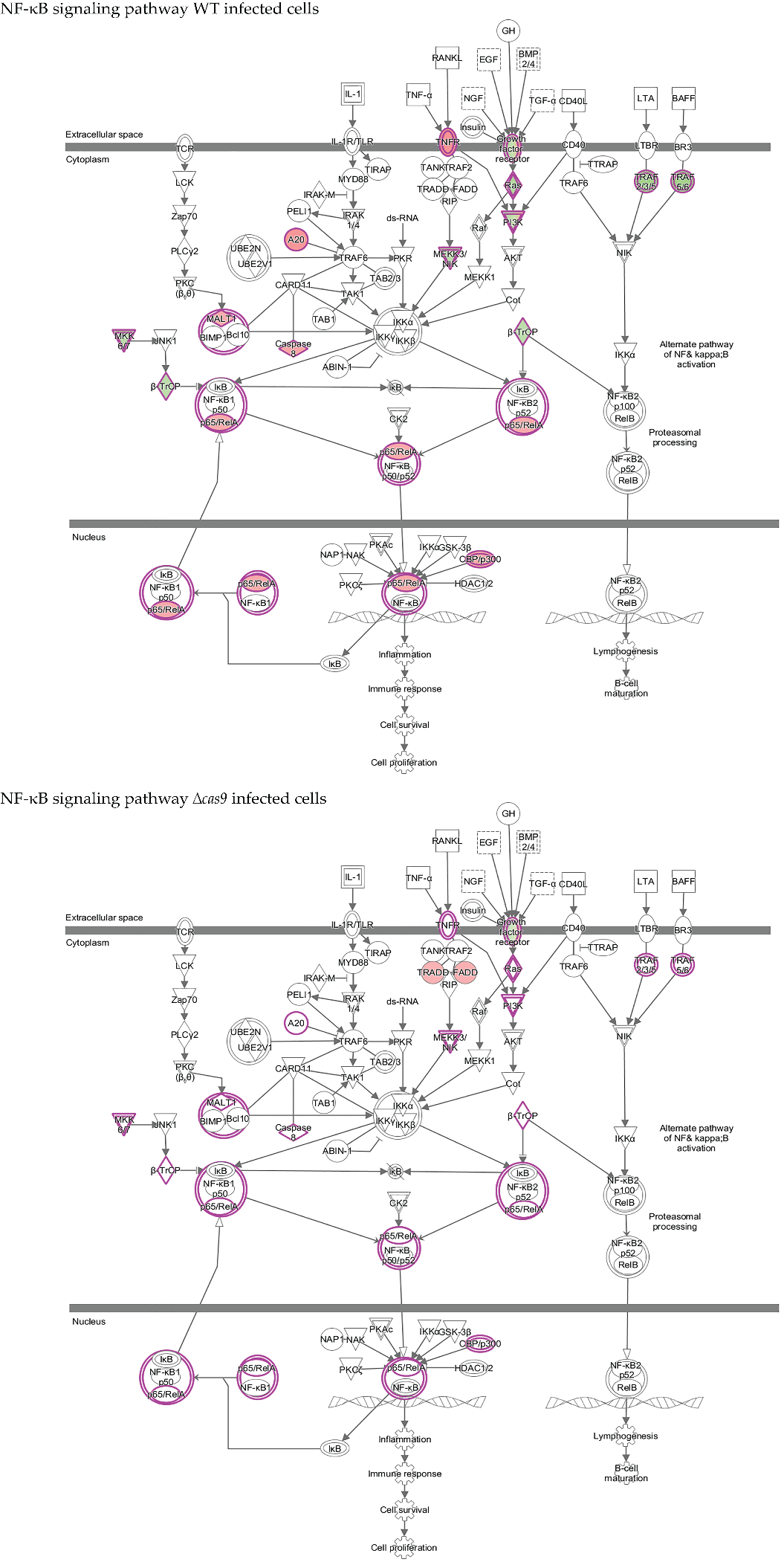


**Figure S3.** IPA shows which Caco-2 NF-κB signaling pathway related genes were significantly modulated upon infection by wild-type Cas9-producing *C. jejuni* strain (WT; upper panel), and by its isogenic *cas9* deletion mutant (∆*cas9*; lower panel). During infection by WT, pro-inflammatory NF-κB pathway genes TNFR, regulator A20, caspase-8, p65(RelA)NF-κB, and CPB/p300 were induced and pathway genes involved in B-cell maturation (alternative pathway of NF-κB activation) were downregulated. The cell proliferation and B-cell maturation parts of the NF-κB pathway were significantly repressed (Table S3). These genes were not differentially expressed during infection by the isogenic ∆*cas9* mutant and the NF-κB pathway was not significantly modulated during infection by this mutant (Figure 4). In the pathway maps, red color represents upregulation of gene expression and green color represents downregulation of gene expression; expression levels are relative to mock-challenged cells.
